# Population-based mammography screening attendance in Sweden 2017–2018: A cross-sectional register study to assess the impact of sociodemographic factors

**DOI:** 10.1016/j.breast.2021.05.011

**Published:** 2021-06-02

**Authors:** Magdalena Lagerlund, Anna Åkesson, Sophia Zackrisson

**Affiliations:** a Department of Translational Medicine, Diagnostic Radiology, Lund University, Skåne University Hospital, 205 02 Malmö, Sweden; b Clinical Studies Sweden – Forum South, Skåne University Hospital, Lund, Sweden

**Keywords:** Mammography, Breast cancer screening, Women's health, Socioeconomic aspects of health

## Abstract

Sweden has a population-based mammography screening programme for women aged 40–74. The objective of this study was to examine the association between mammography screening attendance and sociodemographic factors in 15 of Sweden's 21 health care regions. Register-based information was collected on all mammography screening invitations and attendance during 2017 and 2018, and linked to individual-level sociodemographic data from Statistics Sweden. Odds ratios (ORs) and 95% confidence intervals (CIs) for attendance were computed by sociodemographic factor. The study sample included 1.5 million women, aged 40–75, with an overall screening attendance of 81.3%. The lowest odds of attending were found for women living without a partner (OR = 0.52, 95% CI: 0.52–0.53), low-income women (OR = 0.57, 95% CI: 0.56–0.57), and non-Nordic women born in Europe (OR = 0.60, 95% CI: 0.59–0.61). Other groups with lower odds of attending were women whose main source of income was social assistance or benefits (OR = 0.62, 95% CI: 0.62–0.63), those not owning their home (OR = 0.66, 95% CI: 0.66–0.67), and those with low level of education (OR = 0.72, 95% CI: 0.71–0.73). Having multiple of these sociodemographic characteristics further lowered the odds of attending. Although overall mammography screening attendance in Sweden is high, sociodemographic inequalities exist, and efforts should be made to address these. Particular attention should be given to low-income women who live without a partner.

## Highlights

• Mammography screening attendance in Sweden was 81% in 2017–2018.
• Sociodemographic inequalities were found for screening attendance.
• Having multiple low attendance risk factors further decreased the odds of attending.
• Lowest attendance among low-income women who live without a partner.

## 1 Introduction

European guidelines recommended that women aged 45–74 are screened regularly (i.e. every two to three years depending on age group) in organized mammography screening programmes [1], and most European countries have established national population-based programmes [2]. It is important to understand and address the factors influencing attendance in order to ensure high attendance, which is crucial for the positive effect of these programmes on public health. Furthermore, access to health care services on equal and needs-based terms is at the core of Swedish health care [3].

Nationwide population-based mammography screening has been fully implemented in Sweden since 1997 [4] and includes women aged 40–74 years, based on recommendations from the National Board of Health and Welfare [5]. A national quality register for mammography screening is being established in Sweden, but the historical lack of such a register has prevented the monitoring and reporting of attendance on a national level. Thus, annual mammography screening attendance for Sweden is not available in programme-based health statistics presented by either OECD.stat [6] or Eurostat [7]. An international survey among European countries reported a comparatively high overall attendance of 80% for Sweden in both 2010 and 2014 [2]. However, previous studies have found attendance to be lower in groups who may be socioeconomically vulnerable, such as women who were born abroad [[8], [9], [10], [11]], have low income [[9], [10], [11]], are unmarried or live without a partner [8,[10], [11], [12], [13]], are not gainfully employed [[8], [9], [10],^13^], have lower education, and do not own their home [8]. These studies were limited to two separate health care regions in Sweden (Uppsala and Skåne), and were based on mammography screening data up to 2009.

The objective of this study was to examine the association between mammography screening attendance and sociodemographic factors in 15 of Sweden's 21 health care regions. Attendance by sociodemographic factor are reported.

## 2 Materials and methods

This cross-sectional population-based register study was conducted in Sweden, where women between the ages of 40 and 74 are invited to mammography screening every 18–24 months depending on age and regional capacity. Each health care region individually conducts and administers screening, and there are thus differences in intervals between screening appointments, the layout and content of the invitation letter, hours of operation, ways of cancelling or rescheduling an appointment, reminders, etc. All invitations are sent by post and offer a pre-booked appointment date and time, which does not need to be confirmed, but can be rescheduled or cancelled.

**Fig. 1:**
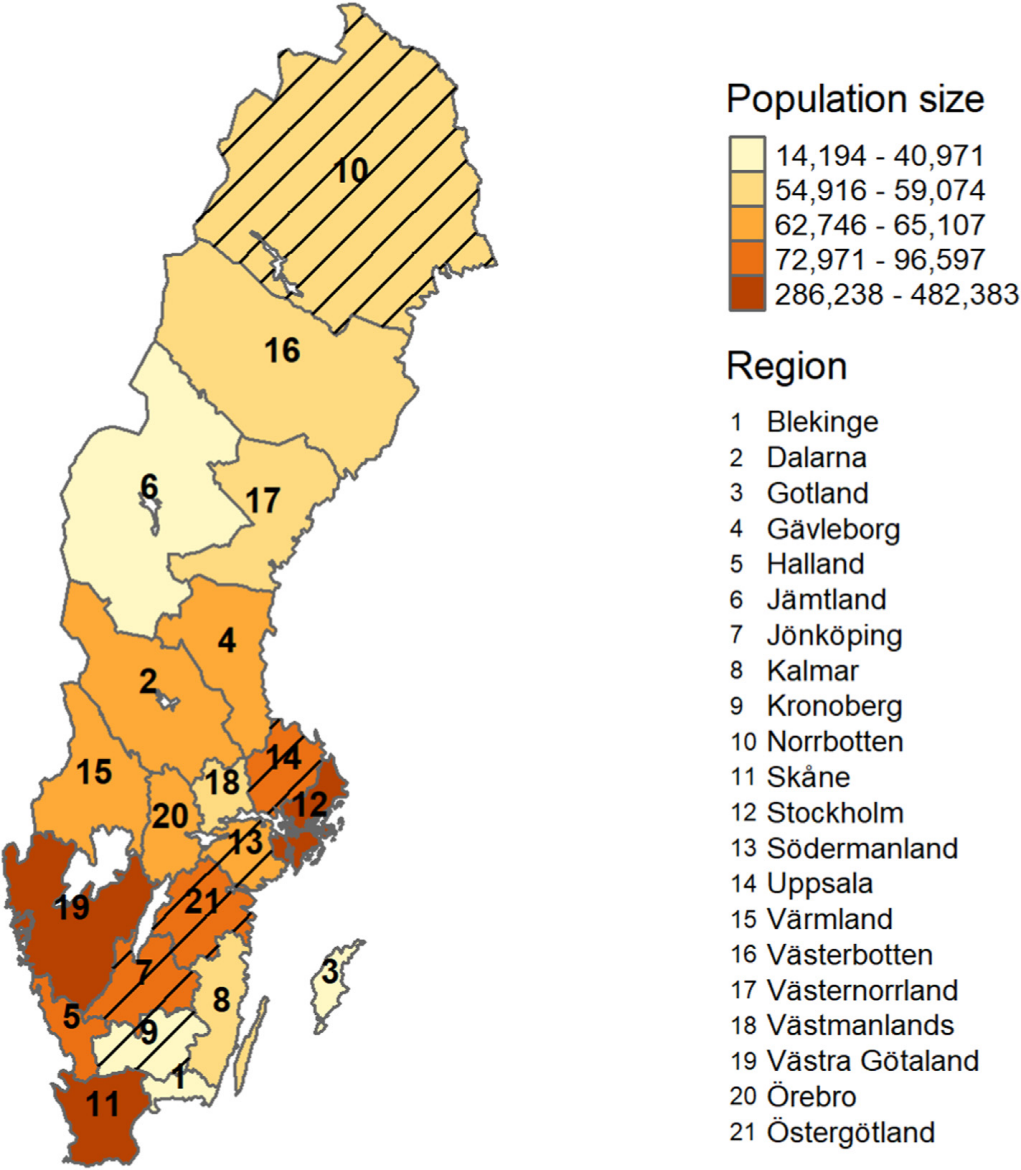
Map of Swedish health care regions showing the population of women aged 40–75 in 2018. Excluded regions are hatched.

Individual screening-related data for all women invited to the screening programme between 2014 and 2018 in 15 of 21 health care regions in Sweden (Fig. 1) were initially extracted, with an original study objective of also comparing attendance before and after removal of the out-of-pocket fee in 2016. These regions include about 81% of the women eligible for mammography screening in Sweden. All these regions used the same company (Sectra AB) for the administration and tracking of invitations, attendance and results throughout the entire study period, which enabled high-quality and consistency of screening data between regions. Among the six regions excluded from this study four (Jönköping, Kronoberg, Norrbotten and Uppsala) used other radiological information systems for all or part of the study period, and two (Sörmland and Östergötland) did not grant us permission to extract data. One of the three programmes operating in Stockholm (Karolinska University Hospital) also did not grant permission. This study was approved by the local ethics committee at Lund University (Nos. 2018/576 and 2018/965). Active informed consent as a requirement for data collection was waived.

The data extracted included the date of the screening appointment, age at the screening appointment, and attendance outcome (attended, cancelled, missed and unavailable), for each regional mammography screening programme separately, and were merged into one dataset. The unique personal identity number assigned to all Swedish residents was used to merge screening data with information on individual-level sociodemographic characteristics obtained from population registers at Statistics Sweden (the Longitudinal Integration Database for Health Insurance and Labour Market Studies (LISA) [14], the Total Population Register [15] and the Geodatabase) [16]. The most recent sociodemographic information for each screening appointment was used. Same-year sociodemographic data were linked to each screening appointment in 2017. In 2018, same-year data were available only for home ownership and type of municipality, and data on income, education and cohabitation from 2017 were used.

**Fig. 2:**
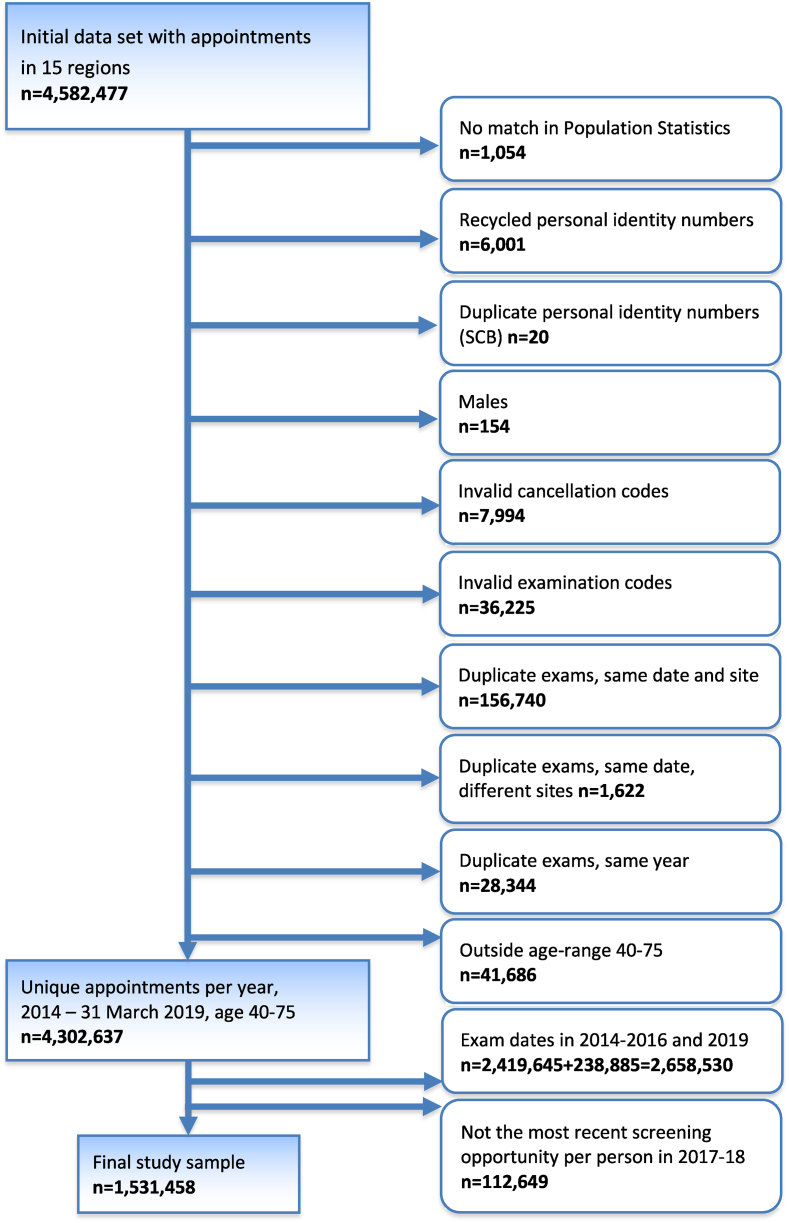
Selection of the final study sample.

The initial dataset included a total of 4,582,477 appointments for 1,780,164 women. After the exclusions described in Fig. 2, the final study sample included 1,531,458 women. Only the most recent screening appointment for each woman, aged 40–75, within the complete two-year period of 2017-18 was included. Women aged 75 were included to allow for a certain overflow from the age limit of 74 years, due to administrative reasons, e.g., rescheduling. Appointments were excluded when personal identity numbers lacked a match, had duplicates or were suspected to have been recycled according to data from Statistics Sweden. Furthermore, appointments with examination or cancellation codes unrelated to mammography screening were excluded, as were appointments where it was known that the invitation did not reach the recipient (unavailable). Duplicate appointments within the same programme, at different locations and within the same year were also excluded.

### 2.1 Outcome variable

The outcome variable was mammography screening attendance (yes/no) at the most recent screening appointment, irrespective of whether it was the original or a rescheduled appointment date, for each woman during the period 2017-18. A two-year period was chosen to allow for longer screening cycles which are common in several health care regions.

### 2.2 Sociodemographic and programme-related variables

Table 1 presents the sociodemographic variables, including age group, cohabitation (in which only couples who had children together were categorized as cohabiting), level of education, income (individual share of equivalized disposable household income in SEK), main source of income, home ownership, country of birth, and type of municipality (based on categorization by the Swedish Association of Local Authorities and Regions) [17]. The programme-related variables included were region and year of screening appointment. Categorizations of data were based on the way in which variables were provided by Statistics Sweden, and by logically and conceptually combining categories without affecting important differences in the attendance outcome, in order to minimize the number of categories. Information on specific country of birth were only retreived for larger groups living in Sweden (n > 20,000 in 2018). Thus, it was necessary to analyze country of birth in larger aggregate categories. Missing data constituted less than 0.3% of the data for all variables except education (1.2%) and home ownership (1.7%), and were excluded from the analyses. All variables were analyzed as categorical variables.

**Table 1:** Sociodemographic and other characteristics of the study sample at the time of the most recent mammography screening appointment in Sweden 2017-18 (N = 1,531,458).

Characteristic	All regions		Stockholm	Other
	n	%	%	%
Mean age (SD)	56.00	(10.09)	54.75	56.37
Age group (years)				
40-44	252,903	16.51	19.80	15.57
45-49	234,095	15.29	16.86	14.83
50-54	242,434	15.83	15.81	15.84
55-59	208,768	13.63	13.47	13.68
60-64	203,443	13.28	12.75	13.44
65-69	197,702	12.91	10.20	13.22
70-75	192,113	12.54	11.11	13.43
Cohabitation (living with partner)				
Yes	906,451	59.19	53.99	60.68
No	620,828	40.54	45.69	39.06
*Missing*	4179	0.27	0.32	0.26
Level of education				
Low (elementary school, ≤9 years)	213,497	13.94	12.34	14.40
Intermediate (secondary school)	673,218	43.96	38.22	45.61
High (post-secondary)	625,993	40.88	47.86	38.87
*Missing*	18,750	1.22	1.58	1.12
Income category				
Lowest (decile 1): ≤98,300 SEK	152,581	9.96	9.11	10.21
Low-medium (deciles 2–4): 98,400–162,700 SEK	458,409	29.93	23.71	31.72
Medium-high (deciles 5–10): ≥162,800 SEK	916,293	59.83	66.86	57.81
*Missing*	4175	0.27	0.32	0.26
Main source of income				
Employment	939,067	61.32	65.32	60.23
Retirement pension	368,554	24.07	20.33	25.14
Student financing	6075	0.40	0.52	0.36
Care of a sick child or relative	8974	0.59	0.84	0.51
Social assistance and benefits:				
Sickness benefit	32,413	2.12	1.94	2.17
Sickness compensation	81,781	5.34	4.16	5.68
Unemployment insurance/benefit	8767	0.57	0.72	0.53
Labour market measure	19,130	1.25	1.42	1.20
Financial assistance	30,033	1.96	2.03	1.94
No income	32,489	2.12	2.60	1.98
*Missing*	4175	0.27	0.32	0.26
Home ownership				
Yes (house or apartment)	1,106,844	72.27	65.65	74.18
No	398,585	26.03	32.38	24.20
*Missing*	26,029	1.70	1.97	1.62
Country of birth				
Sweden	1,213,909	79.26	68.63	82.32
Other Nordic countries	57,226	73.74	5.21	3.31
Other European countries	111,140	7.26	9.68	6.56
Other	149,076	9.73	16.48	7.80
*Missing*	107	0.01	0.01	0.01
Region				
Blekinge	33,110	2.16		
Dalarna	60,682	3.96		
Gotland	13,832	0.90		
Gävleborg	60,775	3.97		
Halland	65,874	4.30		
Jämtland/Härjedalen	26,699	1.74		
Kalmar	50,630	3.31		
Skåne	271,723	17.74		
Stockholm	341,925	22.33		
Värmland	59,993	3.92		
Västerbotten	49,076	3.20		
Västernorrland	50,988	3.33		
Västmanland	47,125	3.08		
Västra Götaland	348,371	22.75		
Örebro	50,655	3.31		
Type of municipality				
Large cities (>200,000)^a^	624,598	40.78	93.22	25.71
Mid-sized cities (50,000–200,000)^b^	472,790	30.87	6.34	37.92
Smaller cities, towns and rural areas	430,520	28.11	0.18	36.14
*Missing*	3550	0.23	0.26	0.22
Year of scheduled appointment				
2017	723,788	47.26	52.99	45.61
2018	807,670	52.74	47.01	54.39

a Includes commuting zone.

b Includes neighbouring municipalities.

### 2.3 Statistical analysis

Odds ratios (ORs) and 95% confidence intervals (CIs) were determined for mammography screening attendance by sociodemographic factor. Unadjusted estimates were calculated, as well as estimates adjusted for the potential confounding effect of region and age group. In multivariable analyses the effect of sociodemographic factors on attendance was further examined, both in the total sample and stratified by region (Stockholm vs. all other regions) and type of municipality.

Combinations of sociodemographic characteristics associated with lower likelihood of mammography screening attendance were also analyzed in order to identify groups with particularly low attendance. The statistical software used for the analyses was SPSS, version 25.

A sensitivity analysis was conducted among the 1,488,224 women whose primary appointment date (i.e., the one set in the invitation) was scheduled for 2017-18, to examine whether the results changed when using an alternative outcome of attendance within 90 days from the primary appointment date. Appointments up to March 31, 2019 were included in the sensitivity analysis. A cut-off of 90 days has been used in other studies examining screening attendance [[18], [19], [20], [21]].

## 3 Results

A total of 1,531,458 women were included in the study sample. The distribution of demographic characteristics is presented in Table 1 and shows that 59% of the study sample lived with a partner, 14% had a low level of education, social assistance and benefits was the main source of income in 11%, and employment or retirement income was the main source of income in 85%, 72% owned their own home, 79% were born in Sweden, 41% lived in a large city or surrounding commuting areas, and 28% lived in smaller cities, towns, or rural areas. The mean age at the time of the screening appointment was 56 years. The regions with the largest proportions of the study sample were Västra Götaland (23%), Stockholm (22%) and Skåne (18%).

Screening attendance per region and by sociodemographic factor are presented in Table 2. The overall attendance was 81.3% for all regions combined. Stockholm had the lowest attendance (71.7%), followed by Örebro (80.7%), while the highest attendance was observed in the northern regions of Västernorrland (87.1%) and Västerbotten (86.7%), and in the southern regions of Blekinge and Halland (86.7%). Screening attendance was lower among women living in large cities (77.0%) than among those living in mid-sized cities (83.7%) or smaller cities, towns or rural areas (85.3%). Attendance according to age group varied from 79.6% among women in their forties, to 84.4% among women 65–69 years of age. The lowest attendance was found among women whose main source of income was social assistance or benefits (66.7%), non-Nordic women born in Europe (68.0%) or elsewhere (68.6%), and among women in the lowest income decile (68.5%). Other sociodemographic groups with attendance below 75% were women who did not own their home (70.8%), women with low level of education (73.8%), and women living without a partner (74.4%).

**Table 2:** Mammography screening attendance in Sweden in 2017–18 by sociodemographic factor.

Variable	Total	Attenders			
	N	All regions		Stockholm	Other
		n	%	%	%
Total	1,531,458	1,244,938	81.29	71.66	84.06
Age group					
40-44	252,903	201,431	79.65	70.93	82.84
45-49	234,095	186,377	79.62	70.35	82.64
50-54	242,434	193,850	79.96	70.11	82.79
55-59	208,768	169,263	81.08	70.93	83.95
60-64	203,443	167,181	82.18	71.08	85.20
65-69	197,702	166,772	84.36	75.13	85.13
70-75	192,113	160,064	83.32	75.53	86.45
Cohabitation					
Yes	906,451	781,575	86.22	77.16	88.54
No	620,828	461,840	74.39	65.46	77.38
Level of education					
Low	213,497	157,628	73.83	60.66	77.08
Intermediate	673,218	550,772	81.81	70.95	84.43
High	625,993	528,040	84.35	76.27	87.21
Income					
Lowest decile	152,581	104,481	68.48	53.09	72.42
Deciles 2-4	458,409	362,576	79.09	66.49	81.80
Deciles 5-10	916,293	776,360	84.73	76.23	87.55
Main source of income					
Employment/retirement	1,307,621	1,100,981	84.20	74.91	86.87
Student financing	6075	4463	73.47	65.82	76.64
Care of sick child/relative	8974	6170	68.75	60.85	72.50
Social assistance/benefits	172,124	114,721	66.65	54.89	69.67
No income	32,489	17,082	52.58	41.12	56.89
Home ownership					
Yes	1,106,844	946,146	85.48	76.84	87.68
No	398,585	282,183	70.80	62.14	74.13
Country of birth					
Sweden	1,213,909	1,023,765	84.34	76.15	86.30
Other Nordic countries	57,226	43,331	75.72	69.42	78.56
Other European countries	111,140	75,561	67.99	59.28	71.68
Other	149,076	102,220	68.57	60.94	73.21
Region					
Blekinge	33,110	28,715	86.73		
Dalarna	60,682	51,165	84.32		
Gotland	13,832	11,368	82.19		
Gävleborg	60,775	50,837	83.65		
Halland	65,874	57,119	86.71		
Jämtland/Härjedalen	26,699	22,963	86.01		
Kalmar	50,630	42,708	84.35		
Skåne	271,723	222,339	81.83		
Stockholm	341,925	245,025	71.66		
Värmland	59,993	51,355	85.60		
Västerbotten	49,076	42,554	86.71		
Västernorrland	50,988	44,425	87.13		
Västmanland	47,125	40,509	85.96		
Västra Götaland	348,371	292,982	84.10		
Örebro	50,655	40,874	80.69		
Type of municipality					
Large cities	624,598	481,078	77.02	72.27	81.98
Mid-sized cities	472,790	395,641	83.68	65.05	84.58
Smaller cities, towns and rural areas	430,520	367,119	85.27	62.28	85.31

The results of the logistic regression analysis of attendance data are presented in Table 3. Generally, the associations between sociodemographic factors and attendance were slightly weakened when adjusting for region and age group, with the exception of education and income, where the association between low and intermediate level of education, as well as the four lower deciles of income, and attendance became stronger. The effect size of having social assistance and benefits as the main source of income also increased. In the multivariable model all associations were reduced compared to both unadjusted estimates and those adjusted for region and age. However, each sociodemographic factor was still independently associated with attendance. The odds of attending were lowest among women who were living without a partner (OR = 0.52, 95% CI: 0.52–0.53), among those with the lowest income (OR = 0.57, 95% CI: 0.56–0.57), and among non-Nordic women born in Europe (OR = 0.60, 95% CI: 0.59–0.61). Furthermore, attendance was less likely among women whose main source of income was social assistance or benefits (OR = 0.62, 95% CI: 0.62–0.63), among those not owning their home (OR = 0.66, 95% CI: 0.66–0.67), and among those with low level of education (OR = 0.72, 95% CI: 0.71–0.73).

**Table 3:** Logistic regression analysis of mammography screening attendance by sociodemographic factor in Sweden in 2017–18, with stratification by region (Stockholm vs. other regions). Odds Ratios (ORs) and 95% Confidence Intervals (CIs).

Variable	Unadjusted	Adjusted for age and region^a^^,^^b^	Multivariate^c^ All regions (N = 1,490,880)	Multivariate Stockholm (N = 330,867)	Multivariate Other regions (N = 1,160,013)
	OR (95% CI)	OR (95% CI)	OR (95% CI)	OR (95% CI)	OR (95% CI)
Cohabitation					
Yes	Ref	Ref	Ref	Ref	Ref
No	0.46 (0.46–0.47)	0.47 (0.46–0.47)	0.52 (0.52–0.53)	0.59 (0.58–0.60)	0.49 (0.49–0.50)
Level of education					
Low	0.52 (0.52–0.53)	0.45 (0.45–0.46)	0.72 (0.71–0.73)	0.70 (0.69–0.72)	0.72 (0.71–0.74)
Intermediate	0.83 (0.83–0.84)	0.76 (0.75–0.77)	0.88 (0.87–0.89)	0.85 (0.84–0.87)	0.89 (0.88–0.90)
High	Ref	Ref	Ref	Ref	Ref
Income					
Lowest decile	0.39 (0.39–0.40)	0.36 (0.36–0.36)	0.57 (0.56–0.57)	0.59 (0.57–0.61)	0.55 (0.54–0.56)
Decile 2-4	0.68 (0.68–0.69)	0.61 (0.60–0.61)	0.74 (0.74–0.75)	0.74 (0.73–0.76)	0.74 (0.73–0.75)
Decile 5-10	Re	Ref	Ref	Ref	Ref
Main source of income					
Employment/retirement	Ref	Ref	Ref	Ref	Ref
Student finance	0.52 (0.49–0.55)	0.58 (0.54–0.61)	0.82 (0.77–0.87)	0.94 (0.86–1.06)	0.75 (0.69–0.81)
Care of sick child/relative	0.41 (0.39–0.43)	0.46 (0.44–0.48)	0.61 (0.58–0.64)	0.70 (0.64–0.75)	0.57 (0.54–0.61)
Social assistance/benefits	0.38 (0.37–0.38)	0.36 (0.35–0.36)	0.62 (0.62–0.63)	0.71 (0.69–0.73)	0.60 (0.59–0.61)
No income	0.21 (0.20–0.21)	0.21 (0.21–0.22)	0.37 (0.36–0.38)	0.40 (0.38–0.42)	0.36 (0.35–0.37)
Home ownership					
Yes	Ref	Ref	Ref	Ref	Ref
No	0.41 (0.41–0.42)	0.43 (0.43–0.44)	0.66 (0.66–0.67)	0.71 (0.70–0.72)	0.65 (0.64–0.65)
Country of birth					
Sweden	Ref	Ref	Ref	Ref	Ref
Other Nordic countries	0.58 (0.57–0.59)	0.61 (0.60–0.62)	0.74 (0.73–0.76)	0.79 (0.76–0.82)	0.72 (0.70–0.74)
Other European countries	0.39 (0.39–0.40)	0.43 (0.42–0.43)	0.60 (0.59–0.61)	0.65 (0.63–0.66)	0.58 (0.57–0.59)
Other	0.41 (0.40–0.41)	0.47 (0.46–0.47)	0.80 (0.79–0.81)	0.76 (0.74–0.77)	0.83 (0.81–0.84)
Age group					
40-44	0.73 (0.71–0.74)	0.77 (0.76–0.78)	0.81 (0.79–0.82)	0.80 (0.77–0.82)	0.81 (0.80–0.83)
45-49	0.72 (0.71–0.74)	0.76 (0.74–0.77)	0.76 (0.75–0.77)	0.76 (0.73–0.78)	0.77 (0.75–0.78)
50-54	0.74 (0.73–0.75)	0.76 (0.75–0.77)	0.76 (0.75–0.78)	0.76 (0.74–0.78)	0.77 (0.75–0.78)
55-59	0.79 (0.78–0.81)	0.81 (0.80–0.82)	0.85 (0.84–0.87)	0.83 (0.80–0.86)	0.87 (0.85–0.88)
60-64	0.86 (0.84–0.87)	0.87 (0.85–0.88)	0.93 (0.92–0.95),	0.84 (0.82–0.87)	0.97 (0.95–1.00)
65-69	Ref	Ref	Ref	Ref	Ref
70-75	0.93 (0.91–0.94)	0.92 (0.90–0.93)	0.97 (0.95–0.99)	1.00 (0.96–1.03)	0.96 (0.94–0.98)
Region					
Blekinge	0.97 (0.93–1.01)	0.97 (0.93–1.01)	1.03 (0.99–1.08)		1.04 (0.99–1.08)
Dalarna	0.79 (0.77–0.82)	0.79 (0.77–0.82)	0.82 (0.79–0.85)		0.82 (0.79–0.85)
Gotland	0.68 (0.65–0.72)	0.68 (0.65–0.71)	0.68 (0.65–0.72)		0.68 (0.65–0.72)
Gävleborg	0.76 (0.73–0.78)	0.75 (0.73–0.78)	0.82 (0.79–0.85)		0.83 (0.80–0.86)
Halland	0.96 (0.93–1.00)	0.97 (0.94–1.01)	0.97 (0.94–1.01)		0.97 (0.94–1.01)
Jämtland/Härjedalen	0.91 (0.87–0.95)	0.91 (0.87–0.95)	0.92 (0.88–0.96)		0.92 (0.88–0.97)
Kalmar	0.80 (0.77–0.83)	0.80 (0.77–0.83)	0.83 (0.80–0.87)		0.83 (0.80–0.87)
Skåne	0.67 (0.65–0.68)	0.67 (0.65–0.69)	0.78 (0.76–0.80)		0.79 (0.76–0.81)
Stockholm	0.37 (0.36–0.38)	0.38 (0.37–0.39)	0.42 (0.41–0.44)		
Värmland	0.88 (0.85–0.91)	0.88 (0.85–0.91)	0.95 (0.92–0.99)		0.96 (0.92–0.99)
Västerbotten	0.96 (0.93–1.00)	0.96 (0.93–1.00)	0.93 (0.90–0.97)		0.93 (0.90–0.97)
Västernorrland	Ref	Ref	Ref		Ref
Västmanland	0.90 (0.87–0.94)	0.91 (0.88–0.94)	1.02 (0.98–1.06)		1.02 (0.98–1.06)
Västra Götaland	0.78 (0.76–0.80)	0.78 (0.76–0.81)	0.87 (0.85–0.90)		0.88 (0.85–0.90)
Örebro	0.62 (0.60–0.64)	0.62 (0.60–0.64)	0.68 (0.66–0.71)		0.69 (0.66–0.71)
Type of municipality					
Large cities	0.58 (0.57–0.58)	0.85 (0.84–0.87)			
Mid-sized cities	0.89 (0.88–0.90)	0.94 (0.93–0.95)			
Smaller cities, towns and rural areas	Ref	Ref			

a Estimates for region are adjusted for age group and estimates for age group are adjusted for region.

b For numbers included please refer to Table 1, All Regions, 2017–2018.

c The multivariate model includes all variables in the table.

A separate analysis was conducted to examine the impact of specific countries of birth on screening attendance (Appendix A). With very few exceptions a statistically significant improvement in the odds of attending was seen for all countries of birth compared to Sweden when adjusting for other sociodemographic factors. Among women born in Iraq and Syria, two of the largest groups in Sweden (1.3% and 1.0% of the study sample, respectively), the odds of attending surpassed those of women born in Sweden after adjusting for other sociodemographic factors. This was also the case for women born in Afghanistan. Other countries were the odds of attending increased by over 100% were African countries, Lebanon and Turkey.

When the multivariable model was stratified by region (Stockholm vs. all other regions) the associations between sociodemographic factors and screening attendance were generally somewhat weaker in Stockholm compared to other regions in Sweden (Table 3). Exceptions were having an intermediate level of education and being born outside of Europe, where women in Stockholm had lower odds of attending mammography screening than women in other regions. Stratification by type of municipality (Table 4) showed that the effect of living without a partner was weaker in large cities, which is partly explained by Stockholm being included in this analysis. The effect of low and intermediate education was weaker in small cities and rural areas. Having an income from other main sources than employment or retirement pension was not as strongly associated with low attendance in large cities, and was more strongly associated with low attendance in small cities and rural areas. The association between country of birth and screening attendance was weaker among women born in a Nordic country and living in large cities, stronger among women born in a European country and living in small cities, and stronger among women born in countries outside of Europe and living in large cities. Stratifying by either region or type of municipality did not change the direction or remove statistical significance for any of the associations between sociodemographic factors and mammography screening attendance.

**Table 4:** Multivariable logistic regression analysis^a^ of mammography screening attendance in Sweden in 2017–18, by sociodemographic factor and stratified by type of municipality. Odds ratios (ORs) and 95% Confidence intervals (CIs).

Variable	Large cities (N = 607,794)	Mid-sized cities (N = 461,921)	Small cities/rural areas (N = 421,165)
	OR (95% CI)	OR (95% CI)	OR (95% CI)
Cohabitation			
Yes	Ref	Ref	Ref
No	0.56 (0.56–0.57)	0.50 (0.49–0.51)	0.48 (0.47–0.49)
Level of education			
Low	0.71 (0.70–0.73)	0.67 (0.65–0.68)	0.77 (0.75–0.80)
Intermediate	0.86 (0.85–0.87)	0.83 (0.82–0.85)	0.98 (0.96–1.00)
High	Ref	Ref	Ref
Income			
Lowest decile	0.57 (0.56–0.59)	0.56 (0.54–0.58)	0.53 (0.52–0.55)
Decile 2-4	0.74 (0.73–0.75)	0.74 (0.72–0.75)	0.74 (0.72–0.76)
Decile 5-10	Ref	Ref	Ref
Main source of income			
Employment/retirement	Ref	Ref	Ref
Student finance	0.90 (0.83–0.98)	0.77 (0.69–0.87)	0.67 (0.59–0.77)
Care of sick child/relative	0.68 (0.63–0.72)	0.55 (0.50–0.61)	0.53 (0.48–0.60)
Social assistance/benefits	0.68 (0.66–0.69)	0.61 (0.60–0.63)	0.56 (0.54–0.57)
No income	0.41 (0.39–0.43)	0.34 (0.32–0.36)	0.34 (0.32–0.36)
Home ownership			
Yes	Ref	Ref	Ref
No	0.67 (0.66–0.68)	0.65 (0.64–0.67)	0.68 (0.67–0.70)
Country of birth			
Sweden	Ref	Ref	Ref
Other Nordic countries	0.81 (0.79–0.84)	0.72 (0.70–0.75)	0.67 (0.64–0.70)
Other European countries	0.62 (0.60–0.63)	0.63 (0.61–0.65)	0.56 (0.54–0.58)
Other	0.73 (0.72–0.75)	0.96 (0.93–0.99)	0.91 (0.88–0.95)

a The model includes all variables in the table, age group and region.

When sociodemographic characteristics, for which the likelihood of attending mammography screening were low (living without a partner, low level of education, lowest income, having social assistance or benefits as the main source of income, not owning one's home and non-Nordic country of birth) were combined in a summary risk factor index, ranging from zero to six (Table 5), attendance declined significantly with each additional risk factor up to four (OR = 0.15, 95% CI: 0.15–0.16), and then increased somewhat. The only specific pairs of risk factors where attendance was lower than for each individual risk factor were low income and living without a partner (OR = 0.23, 95% CI: 0.23–0.24), and living on social assistance and without a partner (OR = 0.30, 95% CI: 0.29–0.30). Only some of the women whose main source of income is social assistance or benefits also fall into the lowest income category (17.9%).

**Table 5:** Mammography screening attendance in Sweden in 2017–18 by sum and specific combinations^a^ of sociodemographic risk factors for low attendance. Odds Ratios (ORs) and 95% Confidence Intervals (CIs).

Variable	Total	Attenders		Adjusted for age and region
	n (%)^b^	n	%	OR (95% CI)
Sum of risk factors^c^				
0 risk factors	554,971 (36.24)	501,109	90.29	Ref
1	470,700 (30.74)	392,860	83.46	0.55 (0.55–0.56)
2	297,837 (19.45)	222,148	74.59	0.33 (0.32–0.33)
3	135,373 (8.84)	85,692	63.30	0.19 (0.19–0.19)
4	53,229 (3.48)	31,162	58.54	0.15 (0.15–0.16)
5	17,570 (1.15)	10,859	61.80	0.17 (0.17–0.18)
6	1778 (0.12)	1108	62.32	0.16 (0.14–0.17)

Low income + living alone	42,674 (2.79)	21,745	50.96	0.23 (0.23–0.24)
Social assistance + living alone	98,524 (6.43)	58,710	59.59	0.30 (0.29–0.30)

a Combinations of two specific risk factors are presented where attendance for the combination of factors is at least ten percent lower than for each individual risk factor.

b Percent of all women (N = 1,531,458).

c Living without a partner, lowest level of education, lowest income level, social assistance, not owning a home and Non-Nordic country of birth.

The sensitivity analysis among women with a primary appointment date in 2017-18 resulted in an attendance of 78.9% within 90 days of the primary appointment date. Compared to the estimates presented in Table 2, attendance was consistently lower across all categories of the sociodemographic variables, and logistic regression estimates were very similar to those presented in Table 3 (data not shown). Among women with a primary appointment date in 2017–18, 43% had rescheduled their appointment either to an earlier date (13%), or a later date (30%) (data not shown). Information on the primary appointment date was missing for 4.9% of women with appointment dates in 2017 and for 1.3% of women with appointment dates in 2018.

## 4 Discussion

In this cross-sectional population-based register study of mammography screening attendance in Sweden in 2017–18, we found that the overall attendance was 81.3%. The lowest odds of attending were found among women living without a partner, low-income women, and non-Nordic women born in Europe. Lower odds of attendance were also observed among women whose main source of income was social assistance or benefits, those not owning their home and among those with low level of education. Having multiple of these sociodemographic characteristics further lowered the odds of attending.

European guidelines state that an attendance over 70% is acceptable, but that an attendance over 75% is desirable [22]. Our study identified unacceptably low attendance among low-income women, women born outside the Nordic countries, and those without employment or retirement pension as the main source of income. Other sociodemographic groups where attendance is below desired level and should be improved are women living without a partner, women with low level of education, and women not owning their home. The markedly lower attendance rate in Stockholm compared to the rest of the country is confirmed by a previous study reporting an attendance rate of 70% in Stockholm in 2012 and historically [20]. The odds of attending did not increase when adjusting for sociodemographic factors in the multivariate analysis, nor did the stratified analysis show stronger associations between sociodemographic factors and screening attendance in Stockholm.

Previous studies have found that socioeconomically vulnerable groups and women born abroad have less favourable breast cancer outcomes and survival rates [[23], [24], [25], [26]], and these factors therefore warrant extra attention. We found that low socioeconomic status, represented by low income, low level of education, not being financially supported mainly by employment or retirement pension, and not being a homeowner, reduced the odds of attending mammography screening. Similar results have been found in previous Swedish and international studies with respect to low income [[9], [10], [11],^27^], employment status [8,9,13,28], low education [9,27], home ownership [8] and area-level socioeconomic deprivation [9,26,29]. However, some studies found no significant effects of employment status [27,30] or socioeconomic level [30], and no, or a U-shaped, association for education [10,11,28,30]. Our finding of lower odds of attending among women born abroad is supported by studies in Sweden [[8], [9], [10], [11]] and Denmark [28], and a review of US studies found lower adherence among recent immigrants [27]. The effect of country of birth (when three aggregate categories were compared to Sweden) was partly accounted for by socioeconomic status, but an independent association with attendance remained, particularly among non-Nordic women born in Europe. In a more detailed analysis of country of birth, women born in Iraq, Syria and Afghanistan were more likely to attend than women born in Sweden once other sociodemographic factors were accounted for. Our findings suggest that the impact of culture, language and socioeconomic factors vary considerably depending on country of birth.

Furthermore, the finding of lower odds of attending among women living without a partner are corroborated by Swedish and international studies, where lower odds of attending were observed among unmarried women [9,10,13,27,30] and among women living without a partner [8,12,28]. Having a partner may increase the probability of being proactive about one's health, perhaps due to feelings of accountability towards a significant other. Cohabitation may also indicate a higher level of social, emotional and practical support as well as household income, although the effect of cohabitation remained stable and statistically significant when adjusting for equivalized disposable income, which takes the whole household income into consideration.

### 4.1 Strengths and limitations

This is the first study in Sweden in which individual-level screening attendance data have been collected from the majority of Sweden's health care regions to assess screening attendance nationally, regionally, and by different sociodemographic factors.

The attendance might be somewhat overestimated since our definition of attendance was based on the appointments taking place within the time period 2017-18, whether it was the primary appointment date offered in the invitation or a rescheduled appointment. Attendance within 90 days of primary appointment dates in 2017-18 was 1.5% points lower. Considering that information on primary appointment date was missing for 4.9% of the women with screening dates in 2017 and for 1.3% of those with screening dates in 2018, attendance based on this date may be slightly less reliable. However, the sensitivity analysis showed that both attendance outcomes had very similar associations with sociodemographic factors.

Not all health care regions in Sweden were included in this study, and the overall attendance presented may not be representative of the whole country. However, the 15 regions included are geographically well distributed and represent populations of all sizes and densities, and we have no reason to believe that regions with particularly high or low attendance would have been systematically selected. Assuming that screening attendance of the excluded programme in Stockholm (Karolinska University Hospital) was also 71.7%, and that the attendance of 84.1% observed among the other 14 regions applied to the six regions not included, yields an estimated overall attendance of 81.3%. When instead, for each of the six excluded regions, imputing the proportion of those attending screening in a neighbouring region, a very similar attendance of 81.4% was obtained. More importantly, it is unlikely that the association between sociodemographic factors and screening attendance would have differed in the excluded regions.

Despite these limitations, our population study has many strengths, including its large size. While many studies that rely on self-reported data may suffer from low response rates and selection bias, the data used in this study for both exposure and outcome measures were obtained from high-quality registers, thus minimizing measurement error and misclassification. However, some women categorized as non-attenders may have attended mammography screening at private clinics. These are more common in the largest cities and this may partially explain the lower attendance in those regions, particularly in Stockholm where there are several private clinics [20].

The sociodemographic data used in this study originate from many different official sources with high coverage [14]. Although complete or almost complete data were available for most variables (age, region, country of birth, income and cohabitation), information on education was missing for 1.2% of the study sample and among 3.6% of the non-attenders (data not shown). An evaluation in 2006 showed that the level and type of education was correct for 85% of individuals, with higher validity among individuals born in Sweden, and it was suggested that the proportion with low education was overestimated [14]. Moreover, information on home ownership was missing for 1.7% of the study sample and 3.3% of non-attenders, and it is plausible that those with missing information are less likely to be homeowners. Thus, we may have underestimated the effect of both education and home ownership on attendance.

Furthermore, when interpreting the effect of cohabitation, it is important to consider that common-law partners cannot be linked in Swedish official records on households if they do not have children together. Therefore, the number of women cohabiting is somewhat underestimated. This classification bias would likely have attenuated rather than inflated the effect of cohabitation on screening attendance.

## 5 Conclusions

Significant variations in the likelihood of attending mammography screening were found for cohabitation, education, income level and main source, home ownership and country of birth. Having combinations of these characteristics further decreased the odds of attending. Although overall attendance in Sweden is high, continuous efforts should be made to address persisting inequalities. Particular attention should be given to low-income women who live without a partner. These finding may apply to similar settings with population-based screening, universal health care, and a high screening uptake.

## Declaration of competing interest

None.

## Figures and Tables

**Table:** 

Country	Total (N)	Proportion of sample (%)	Attended (N)	Attended (%)	OR (95% CI)	Adjusted OR (95% CI)^a^	Increase in OR (%)
Nordic countries							
Sweden	1,213,909	79.26	1,023,765	84.34	Ref	Ref	
Denmark	8220	0.54	6177	75.15	0.56 (0.53–0.59)	0.71 (0.67–0.76)	26.87
Finland	38,435	2.51	29,219	76.02	0.59 (0.58–0.60)	0.77 (0.75–0.79)	30.05
Norway	9501	0.62	7142	75.17	0.56 (0.54–0.59)	0.70 (0.66–0.74)	24.56
Nordic other	1070	0.07	793	74.11	0.53 (0.46–0.61)	0.63 (0.54–0.73)	17.67

European Union (28)							
Germany	7421	0.48	5028	67.75	0.39 (0.37–0.41)	0.48 (0.45–0.50)	22.31
Poland	21,062	1.38	13,217	62.75	0.31 (0.30–0.32)	0.48 (0.47–0.50)	53.67
Romania	5060	0.33	3179	62.83	0.31 (0.30–0.33)	0.46 (0.43–0.49)	46.18
United Kingdom	2856	0.19	2086	73.04	0.50 (0.46–0.55)	0.64 (0.58–0.70)	26.44
EU28 other	23,543	1.54	15,817	67.18	0.38 (0.37–0.39)	0.57 (0.56–0.59)	51.05

Other European countries							
Bosnia and Herzegovina	11,940	0.78	8705	72.91	0.50 (0.48–0.52)	0.68 (0.65–0.71)	36.20
Yugoslavia	16,511	1.08	11,969	72.49	0.49 (0.47–0.51)	0.69 (0.67–0.72)	41.72
Russia	4860	0.32	3237	66.60	0.37 (0.35–0.39)	0.62 (0.58–0.67)	68.65
Europe other	8046	0.53	5766	71.66	0.47 (0.45–0.49)	0.83 (0.79–0.88)	76.60

USA	2714	0.18	1780	65.59	0.35 (0.33–0.38)	0.49 (0.45–0.54)	38.14
North America other	3164	0.21	2216	70.04	0.43 (0.40–0.47)	0.71 (0.65–0.77)	62.90

Chile	6815	0.45	4512	66.21	0.36 (0.35–0.38)	0.66 (0.63–0.70)	81.32
South America other	8657	0.57	6089	70.34	0.44 (0.42–0.46)	0.77 (0.73–0.81)	74.77

Africa							
Eritrea (East Africa)	4249	0.28	2810	66.13	0.36 (0.34–0.39)	1.01 (0.94–1.08)	178.24
Ethiopia (East Africa)	3573	0.23	2142	59.95	0.28 (0.26–0.30)	0.63 (0.59–0.68)	126.62
Somalia (East Africa)	6377	0.42	2639	41.38	0.13 (0.13–0.14)	0.37 (0.35–0.39)	180.15
Africa other	10,758	0.70	7082	65.83	0.36 (0.34–0.37)	0.79 (0.76–0.83)	120.39

Oceania	592	0.04	405	68.41	0.40 (0.34–0.48)	0.59 (0.48–0.71)	45.52

Asia							
Afghanistan	3737	0.24	2664	71.29	0.46 (0.43–0.50)	1.19 (1.09–1.29)	157.70
China	4286	0.28	2998	69.95	0.43 (0.41–0.46)	0.77 (0.72–0.83)	78.70
India	2792	0.18	1933	69.23	0.42 (0.39–0.45)	0.62 (0.57–0.68)	47.85
Iran	13,791	0.90	10,104	73.27	0.51 (0.49–0.53)	0.83 (0.80–0.87)	63.06
Iraq	19,807	1.29	14,102	71.20	0.46 (0.45–0.47)	1.08 (1.04–1.12)	135.29
Lebanon	5563	0.36	3863	69.44	0.42 (0.40–0.45)	0.89 (0.83–0.94)	109.95
Syria	15,003	0.98	10,659	71.05	0.46 (0.44–0.47)	1.15 (1.10–1.19)	151.54
Thailand	13,290	0.87	9494	71.44	0.47 (0.45–0.48)	0.83 (0.80–0.87)	78.28
Turkey	9841	0.64	6557	66.63	0.37 (0.36–0.39)	0.84 (0.80–0.88)	126.15
Asia other	22,266	1.45	15,694	70.48	0.44 (0.43–0.46)	0.74 (0.71–0.76)	65.54

Soviet Union	1617	0.11	1015	62.77	0.31 (0.28–0.35)	0.49 (0.44–0.55)	57.83

Stateless	2	0.00	1	50.00	0.19 (0.01–2.97)	0.30 (0.01–6.76)	62.37
Unknown	23	0.00	18	78.26	0.67 (0.25–1.80)	0.96 (0.34–2.73)	43.80
Missing	107	0.01	61	57.01	0.25 (0.17–0.36)	0.58 (0.37–0.91)	134.96

a Adjusted for age, region, cohabitation, education, income, main source of income and home ownership.
